# OSM-9 and an amiloride-sensitive channel, but not PKD-2, are involved in mechanosensation in *C*. *elegans* male ray neurons

**DOI:** 10.1038/s41598-018-25542-1

**Published:** 2018-05-08

**Authors:** Hu Zhang, Xiaomin Yue, Hankui Cheng, Xiaoyan Zhang, Yang Cai, Wenjuan Zou, Guifang Huang, Lufeng Cheng, Fang Ye, Lijun Kang

**Affiliations:** 1 Department of Neurobiology, Institute of Neuroscience, Key Laboratory of Medical Neurobiology of the Ministry of Health of China, Zhejiang University School of Medicine, Hangzhou, China; 2 Department of Pharmacology, Basic Medical College, Xinjiang Medical University, Urumqi, China; 3 Department of Immunology, School of Preclinical Medicine, Guangxi Medical University, Nanning, China

## Abstract

Mechanotransduction is crucial for touch sensation, hearing, proprioception, and pain sensing. In *C*. *elegans*, male ray neurons have been implicated to be involved in the mechanosensation required for mating behavior. However, whether ray neurons directly sense mechanical stimulation is not yet known, and the underlying molecular mechanisms have not been identified. Using *in vivo* calcium imaging, we recorded the touch-induced calcium responses in male ray neurons. Our data demonstrated that ray neurons are sensitive to mechanical stimulation in a neurotransmitter-independent manner. PKD-2, a putative sensor component for both mechanosensation and chemosensation in male-specific neurons, was not required for the touch-induced calcium responses in RnB neurons, whereas the TRPV channel OSM-9 shaped the kinetics of the responses. We further showed that RnB-neuron mechanosensation is likely mediated by an amiloride-sensitive DEG/ENaC channel. These observations lay a foundation for better understanding the molecular mechanisms of mechanosensation.

## Introduction

Mechanotransduction is crucial for touch sensation, hearing, proprioception, and pain^1,2^. At the molecular level, four classes of ion channels have been considered as mechano-electrical transduction channels in the animal kingdom: the touch-sensitive ENaC family of Na^+^ channels in *C*. *elegans* (MEC-4/MEC-10 and DEG-1), the stretch-sensitive two-pore domain K^+^ channels (TREK-1/TRAAK), the N-type transient receptor potential (TRP) channel (TRPN1/TRP-4/NOMPC), and piezo proteins^2–6^. Recently, an additional class of membrane proteins (Transmembrane channel-like proteins, TMC) have also been linked to mechanotransduction in vertebrate hair cells^7,8^. However, because of the difficulties associated with functionally reconstituting mechanotransduction channels in heterologous systems, the molecular identities of a vast majority of mechanotransduction channels remain poorly understood^2,4,9^.

*C*. *elegans* has two sexual forms, which include hermaphrodites and males. Male mating facilitates the exchange of genetic material and is evolutionarily beneficial^10^. Male mating behavior has been considered to be one of the most complex behaviors in *C*. *elegans*, which relies on both chemosensation and mechanosensation^11,12^. *C*. *elegans* males have unique tail fans and a hook used during mating. The male tail fan consists of a cuticle and 18 rays. Each ray is composed of a single structural cell and two morphologically distinct sensory neurons, including type A ray neurons (or ray A-neurons, termed RnA neurons, which number from 1–9) and type B ray neurons (or ray-B neurons, termed RnB neurons, which number from 1–9)^12–14^. These ray neurons likely act as mechanical and chemical sensors to detect the proximity of or contact with a hermaphrodite during mating^12,15^. Nevertheless, whether ray neurons directly sense mechanical stimulation is not well-understood, and the underlying molecular mechanisms have yet to be identified.

The TRP channel proteins, LOV-1 (TRPP1) and PKD-2 (TRPP2), are expressed in all RnB neurons (except R6B) and they are required for male mating behavior^16,17^. Males with loss-of-function mutations in *pkd-*2 display significantly impaired responses to hermaphrodite contact and vulva identification^16,17^. In vertebrates, depletion of the polycystin orthologs PKD1 and/or PKD2 may lead to an impairment of flow sensing in the primary cilium of renal epithelial cells in nephrons^18–20^. Thus, PKD-2 has been speculated to be part of the sensory receptor complex mediating mechanosensation in male-specific neurons^12^.

In this study, we employ *in vivo* calcium imaging to monitor touch-evoked activities in male ray neurons. We demonstrate that ray neurons are sensitive to mechanical stimulation in a cell-autonomous manner. The transient receptor potential (TRP) vanilloid channel subunit OSM-9, but not PKD-2, is involved in mechanical signal transduction in RnB neurons. We further show that amiloride blocks touch-induced calcium increases in RnB neurons, suggesting that amiloride-sensitive sodium channel(s) (ENaCs) are likely the primary mechanotransduction channels in RnB neurons.

## Results

### RnB neurons are sensitive to mechanical stimulation

**Figure 1:**
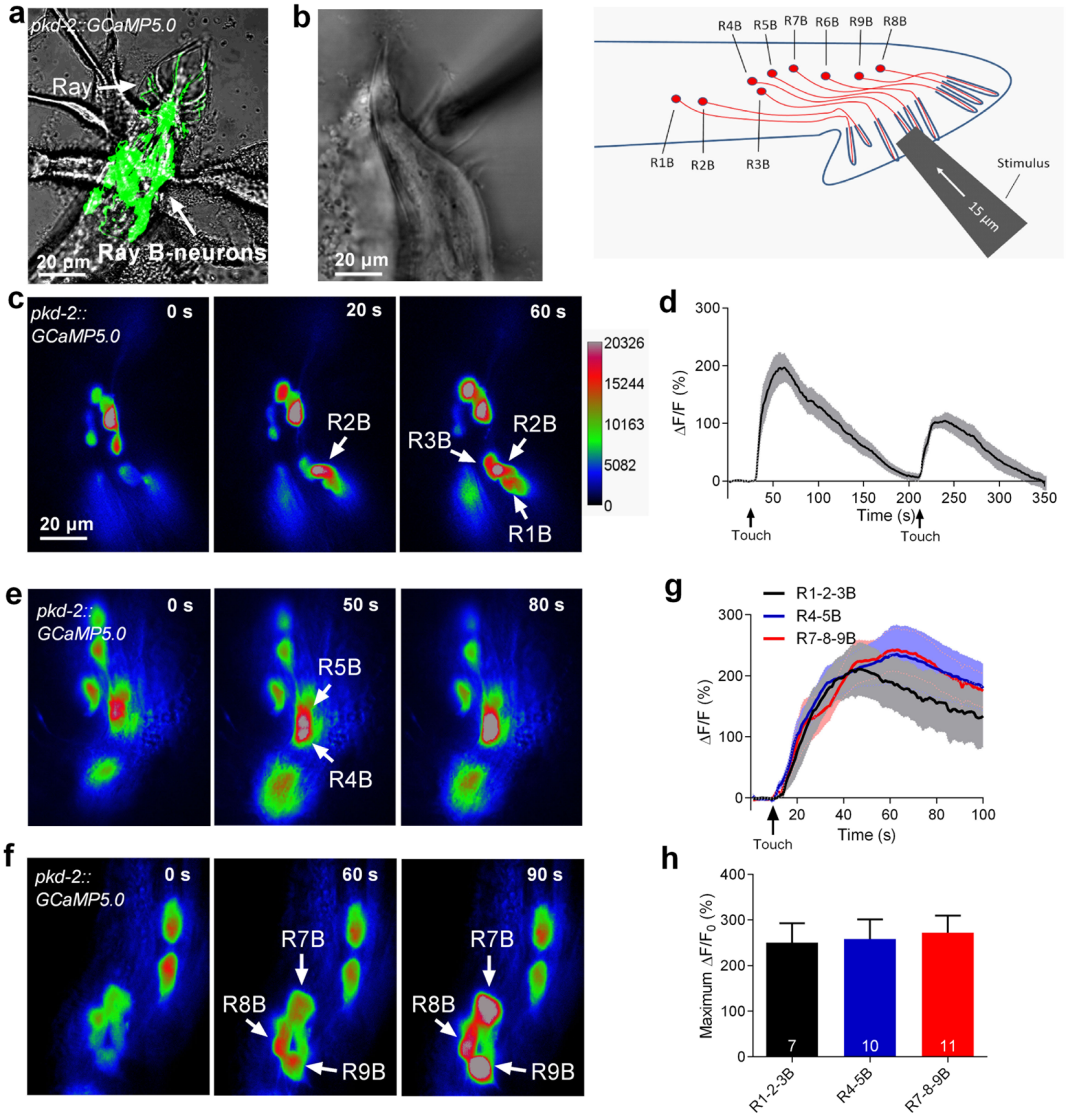
Touch-induced calcium responses in RnB neurons. (**a**) Micrograph of the male tail showing the expression of *pkd-2::GCaMP5*.*0* in the rays. All RnB neurons, except R6B, express GCaMP5.0 by the control of the *pkd-2* promoter. (**b**) A schematic illustrating of delivering mechanical stimulation toward the RnB cilia (at the position of rays 4–6 is shown). Worms expressing GCaMP5.0 in RnB neurons were immobilized with glue and immersed in a bath solution. (**c**) Representative time-lapse rainbow images of GCaMP5.0 based calcium responses from R1B- R3B neurons induced by mechanical stimulation of 15 μm displacement at the position of rays 1–3. (**d**) Calcium responses of the R1B- R3B neurons induced by two successive mechanical stimuli of 15 μm displacement with 180 s interval at the position of rays 1–3. Solid lines show the average fluorescence changes and the shading indicates SEM. n = 10. (**e**) Representative time-lapse rainbow images of GCaMP5.0 based calcium responses from R4B and R5B induced by mechanical stimulation of 15 μm displacement at the position of rays 4, 5. (**f**) Representative time-lapse rainbow images of GCaMP5.0 based calcium responses GCaMP5.0 based calcium responses from R7B- R9B neurons induced by mechanical stimulation of 15 μm displacement at the position of rays 7–9. (**g**,**h**) Calcium responses (**g**) and maximum ΔF/F0 changes (**h**) of the RnB neurons in response to mechanical stimulation. Solid lines show the average fluorescence changes and the shading indicates SEM. n ≥ 7. All error bars represent SEM.

To determine whether RnB neurons respond to mechanical stimulation, a genetically encoded calcium indicator, GCaMP5.0, was expressed in all RnB neurons (except R6B) under the control of the *pkd-2* promoter (Fig. 1a)^21,22^. A glass probe was used to exert a mechanical stimulus, while the fluorescence changes were recorded (Fig. 1b). Using this method, we observed dramatic touch-induced calcium increases in R1B, R2B, and R3B neurons when a mechanical stimulation consisting of a 15-μm displacement was applied to the region of rays 1/2/3 (Fig. 1c). The calcium levels in these neurons were recovered minutes later, and could rise up again when we gave them another mechanical stimulation (Fig. 1d, S1 and Movie S1). Similarly, touch-induced calcium increases were observed in the R4B, R5B, R7B, R8B, and R9B neurons when we stimulated rays 4/5 and rays 7/8/9, respectively (Fig. 1e,f). Notably, no statistical differences in either the amplitude or kinetics of calcium increases in the various ray B neurons were observed when the touch probe moved forward to the indicated rays (Fig. 1g,h). These results demonstrate that RnB neurons are sensitive to mechanical stimulation.

### RnA neurons occasionally respond to mechanical stimulation

**Figure 2:**
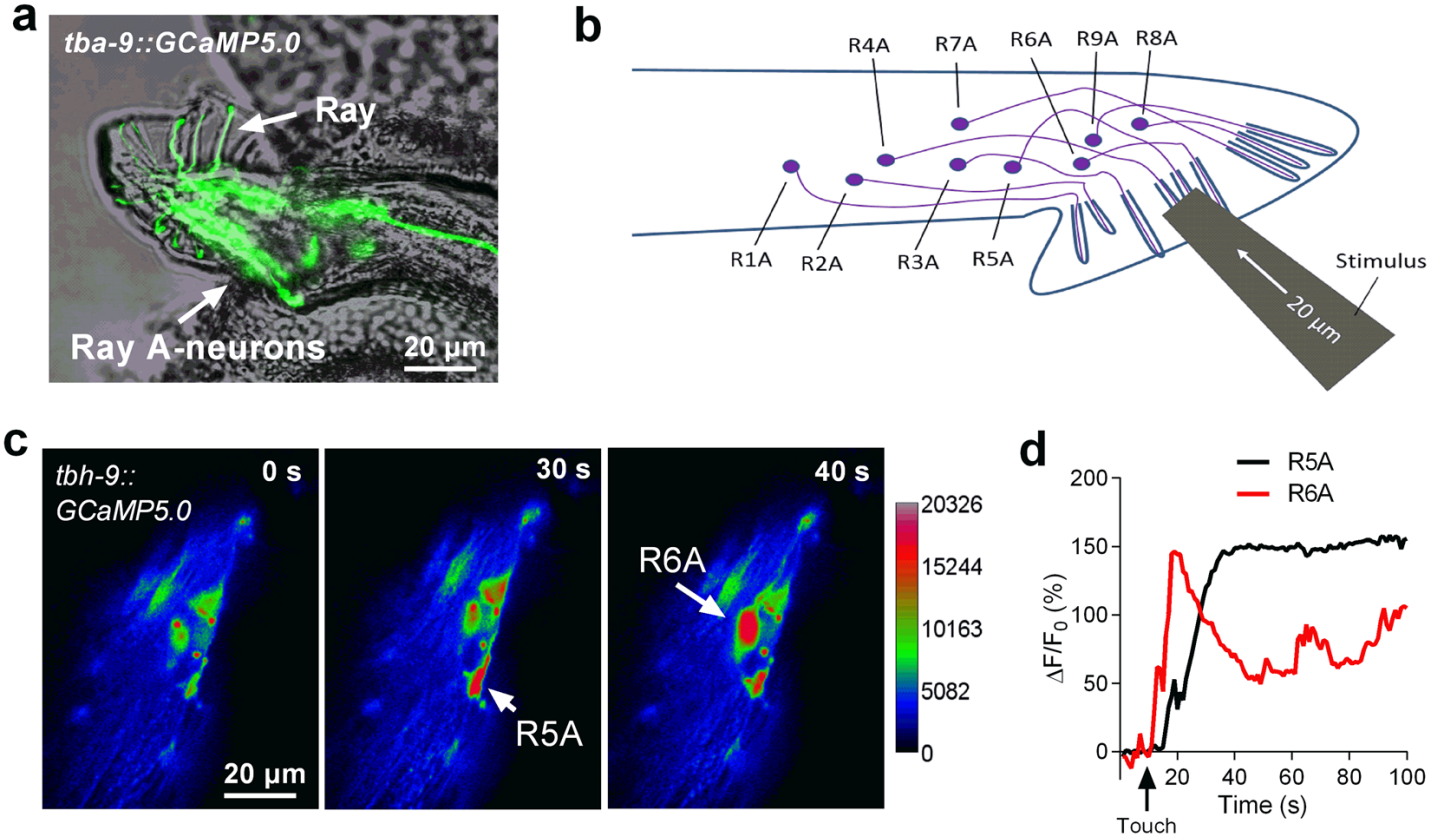
Touch-induced calcium responses in RnA neurons. (**a**) Micrograph of the male tail showing the expression of *tba-9::GCaMP5*.*0* in the rays. (**b**) A schematic illustrating of delivering mechanical stimulation toward the RnA cilia (at the position of rays 4–6 is shown). (**c**,**d**) Representative time-lapse rainbow images of GCaMP5.0 based calcium responses (**c**) and soma fluorescence changes (**d**) from R5A and R6A neurons induced by mechanical stimulation of 20 μm displacement at the position of ray5 and ray6.

We next asked whether RnA neurons respond to mechanical stimulation. We expressed GCaMP5.0 in RnA neurons under the control of the *tba-9* promoter (Fig. 2a)^23^. We speculated that RnA neurons might be much more sensitive to mechanical stimulation than RnB neurons because TRP-4, a pore-forming subunit of a gentle nose touch-related mechano-gated channel, is expressed in some RnA neurons^4,12,24,25^_._ Surprisingly, no detectable calcium response was observed in any RnA neuron when a mechanical stimulation consisting of a 15-μm displacement was applied. A mechanical stimulation exceeding a 20-μm displacement occasionally induced calcium increases in some RnA neurons (4 out of 20 worms) (Fig. 2c,d). These results suggest that RnA neurons may also be activated by mechanical stimulation, but their sensitivity is quite low in our experimental setting. We next focused our study on the touch-induced responses of RnB neurons (particularly calcium responses in R1B, R2B, and R3B neurons [R1B–R3B]) induced by a mechanical stimulation consisting of a 15-μm displacement applied to the region of rays 1–3.

### Touch-induced calcium responses in RnB neurons do not rely on synaptic transmission

**Figure 3:**
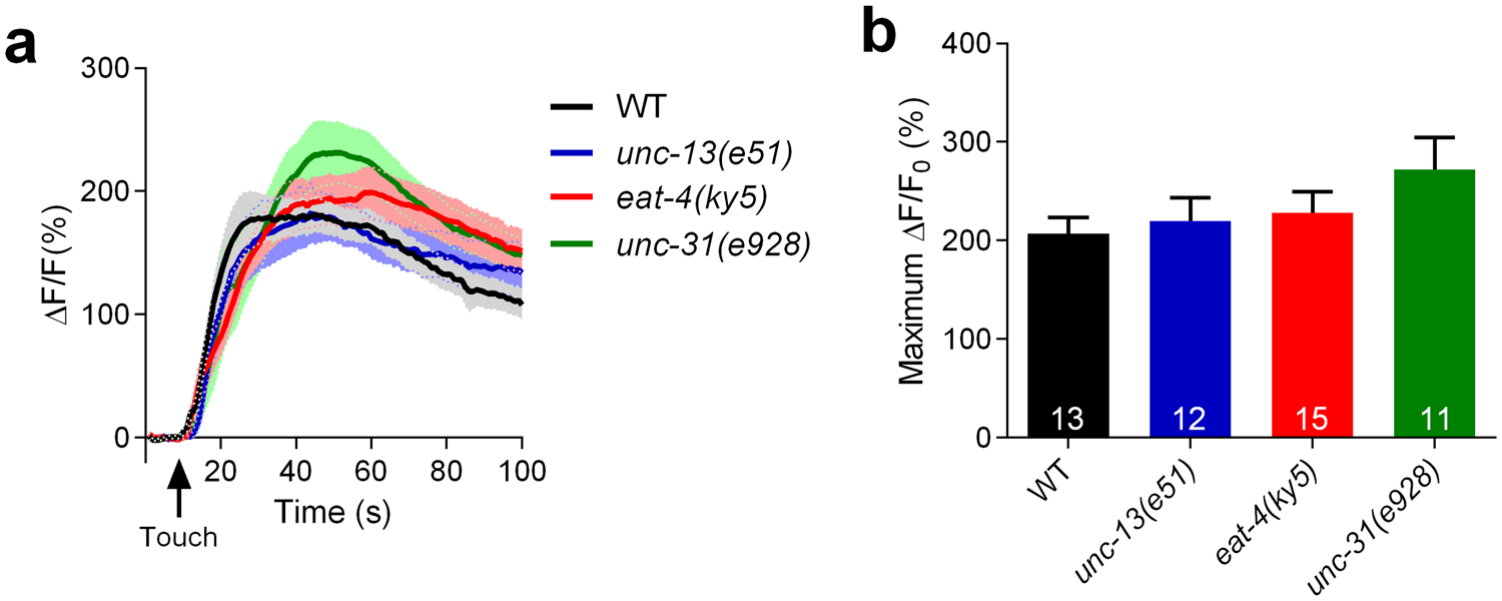
Touch-induced calcium responses in RnB neurons do not rely on synaptic transmission. (**a**,**b**) Averaged calcium responses (**a**) and maximum ΔF/F0 changes (**b**) in R1B-R3B neurons induced by mechanical stimulation of 15 μm displacement at the position of rays 1–3 in wild type, *unc-13(e51)* mutants, *unc-31(e928)* mutants and *eat-4(ky5)* mutants. Solid lines show the average fluorescence changes and the shading indicates SEM. n ≥ 12. Data are mean ± SEM.

One possibility is that calcium increases in RnB neurons following mechanical stimulation are post-synaptically induced by other neurons. Therefore, we examined the touch-induced responses of RnB neurons in *unc-13(e*5*1)*, *eat-4(ky5)*, and *unc-31(e928)* mutant worms. Specifically, *unc-13* and *unc-31* encode orthologs of the mammalian Munc13 and CAPS proteins, which are required for neurotransmitter and neuropeptide release, respectively^26,27^. Additionally, *eat-4* encodes an ortholog of the mammalian vesicular glutamate transporter, which is necessary for glutamatergic neurotransmission^28^. Interestingly, touch-induced calcium increases in RnB neurons in mutants for *unc-13*, *unc-31*, or *eat-4* were similar to those of wild-type worms, suggesting that RnB neurons are likely the primary neurons for sensing mechanical stimulation (Fig. 3a,b).

### PKD-2 is not involved in mechanotransduction in RnB neurons

**Figure 4:**
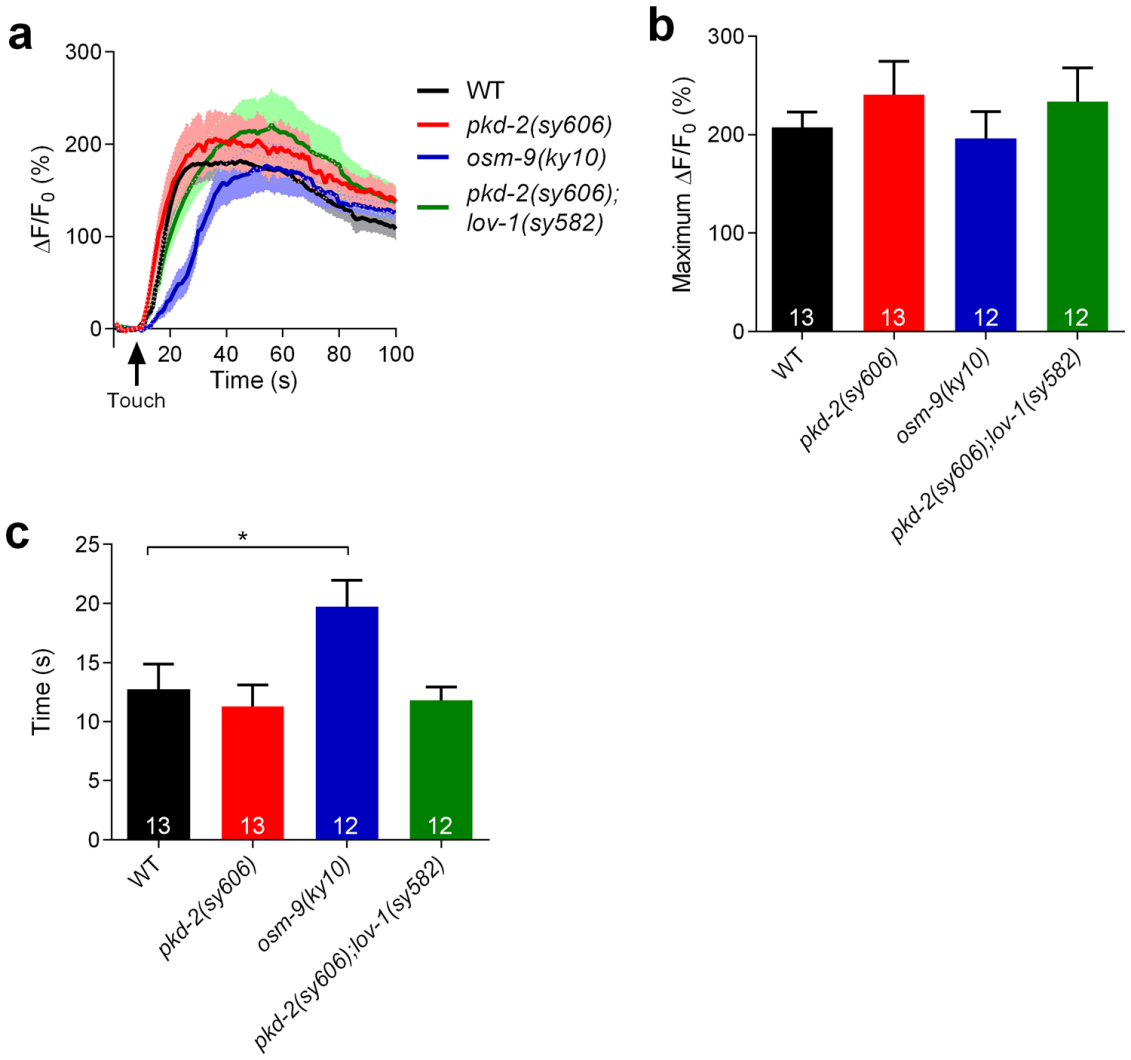
OSM-9, but not PKD-2, is involved in touch-induced calcium responses in RnB neurons. (**a**,**b**) Averaged calcium responses (**a**) and maximum ΔF/F0 changes (**b**) in R1B-R3B neurons induced by mechanical stimulation of 15 μm displacement at the position of rays 1–3 in wild type, *pkd-2(sy606)* mutants, *osm-9(ky10)* mutants, and *pkd-2(sy606);lov-1(sy582)* double mutants. Solid lines show the average fluorescence changes and the shading indicates SEM. (**c**) Half-maximum response times of touch induced-calcium responses in R1B-R3B neurons in wild types, *pkd-2(sy606)* mutants, *osm-9(ky10)* mutants, and *pkd-2(sy606);lov-1(sy582)* double mutants. n ≥ 12. Data are mean ± SEM. *P < 0.05, unpaired Student’s t-tests.

We next sought to investigate the molecular mechanisms of mechanotransduction in RnB neurons. PKD-2 has been implicated in contact responses of adult male towards hermaphrodites. Thus, PKD-2 has been speculated to be part of the sensory receptor complex mediating chemosensation and/or mechanosensation in male-specific neurons^12,17^. Surprisingly, we found that touch-induced calcium responses in neither *pkd-2(sy606)* mutants nor *pkd-2(sy606);lov-1(sy582)* double mutants were impaired (Fig. 4a,b), strongly suggesting that PKD-2 is not involved in mechanotransduction in RnB neurons.

### OSM-9 is involved in mechanosensation of RnB neurons

The TRPV channel subunits of OSM-9 are required in the ASH sensory neurons for avoidance responses to nose touches and aversive chemicals^29^. In adult males, OSM-9 has been reported to be expressed in male-specific neurons in the tail (possibly in the HoB and RnB neurons) and in the male-specific CEM neurons in the head^30,31^. Furthermore, OSM-9 is required for male sexual attraction behaviors^31^. We found that *osm-9(ky10)* mutant males have normal touch-induced calcium increases in RnB neurons (Fig. 4a,b). However, touch-induced calcium increases in RnB neurons in *osm-9(ky10)* mutants were significantly slower than in wild type animals (Fig. 4c). OSM-9::GFP has been previously reported to localize to the endoplasmic reticulum (ER) of the cell body, but not to the cilia of RnB neurons^30^. Taken together, OSM-9 may act downstream of the primary mechanotransduction channel as a calcium modulator in RnB neurons. It should be noted that we did not observe a deficit in contact responses in *osm-9* mutants, probably because of the minor role of OSM-9 in mechanosensation of RnB neurons.

### Amiloride-sensitive channel(s) mediates the mechanosensation of RnB neurons

**Figure 5:**
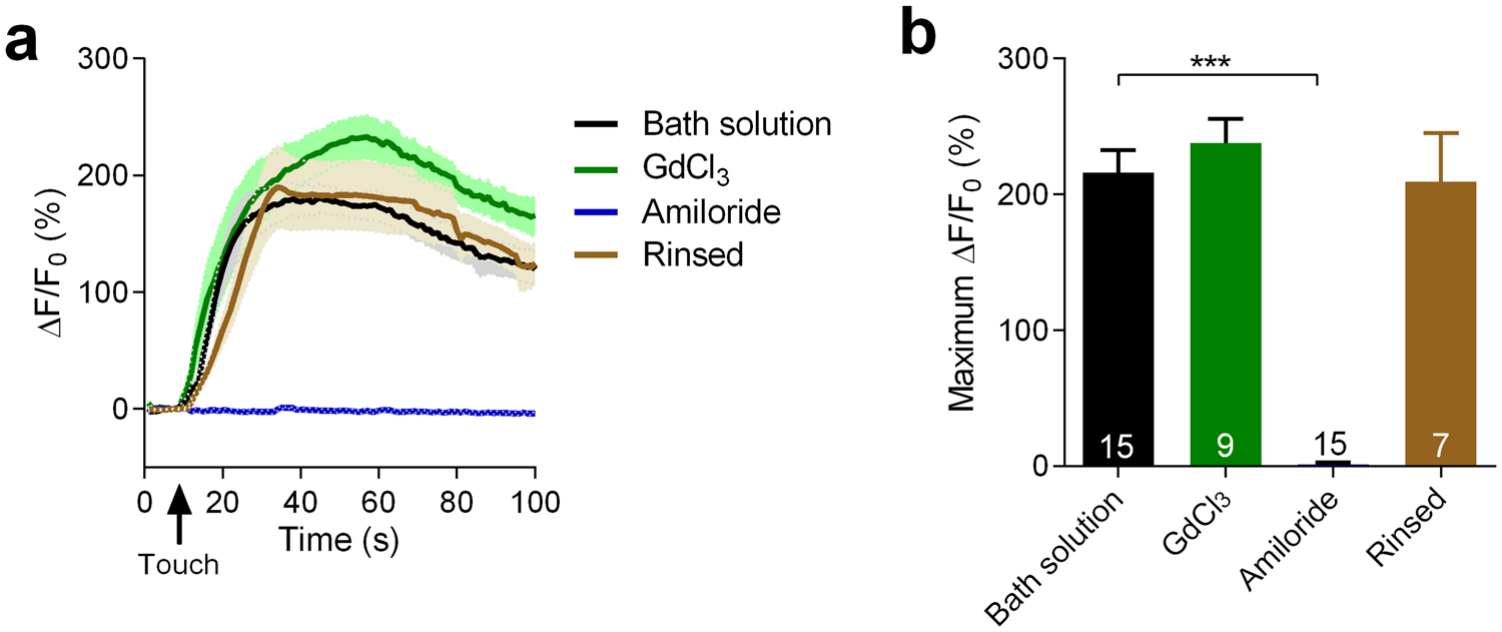
Amiloride blocks touch-induced calcium responses in RnB neurons. (**a**,**b**) Averaged calcium responses (**a**) and maximum ΔF/F0 changes (**b**) in R1B-R3B neurons induced by mechanical stimulation of 15 μm displacement at the position of rays 1–3 in wild type worms with bath solution, GdCl_3_, amiloride or after rinsed amiloride out with bath solution. Solid lines show the average fluorescence changes and the shading indicates SEM. n ≥ 7. Data are mean SEM. ***P < 0.001, unpaired Student’s t-test.

To further characterize the molecular identity of the mechanotransduction channel in RnB neurons, we tried two cation channel blockers, including the DEG/ENaC channel blocker amiloride and a non-specific cation channel blocker GdCl_3_^2,32^. Interestingly, touch-induced calcium increases were fully eliminated by 200 μM amiloride in most RnB neurons except R3B, and recovered after amiloride was rinsed out (Fig. 5a,b). By contrast, touch-induced calcium increases in all RnB neurons were not affected by 100 μM GdCl_3_ (Fig. 5a,b). These results suggest that a DEG/ENaC channel, but not a GdCl_3_-sensitive cation channel, is likely the basic component of the mechanotransduction channel in RnB neurons.

## Discussion

*C*. *elegans* male ray neurons have long been considered candidate mechanosensory neurons^12,15,33^. However, clear evidence showing that ray neurons directly respond to mechanical stimulation has been lacking. Here, we demonstrate that ray neurons can be activated by mechanical stimulation in a cell-autonomous manner. Our data further show that OSM-9 and an amiloride-sensitive channel, but not PKD-2, are required for mechanosensation in RnB neurons.

Whether TRP family channels function as primary mechanotransduction channels has long been of great interest^9^. Recently, TRPN proteins (TRPN1/NOMPC/TRP-4) were confirmed to be cilia-associated mechano-gated channels in both *C*. *elegans* and flies^4,5^. The unusually long N-terminal repeat, which consists of 28 ankyrin domains of the TRPN subunit, presumably acts as the gating spring by which force induces channel gating^34^. Nevertheless, TRPN proteins appear to have been lost in vertebrates^35^. Importantly, there is no evidence showing that any of the other TRP proteins are mechanically gated, even though many members of the TRP subfamily proteins have been implicated in mechanosensation^4,9,36^. PKD-2 has also been considered a strong candidate mechanotransduction channel in RnB neurons because its ortholog is likely to function in flow sensation in the primary cilium of human renal epithelial cells^12,18^. Strikingly, our data show that *pkd-2* mutant worms have no detectable defects in touch-induced calcium responses in RnB neurons, excluding the role of PKD-2 in mechanotransduction in RnB neurons.

Our study also demonstrates that mechanotransduction in RnB neurons is mediated by amiloride-sensitive channels. These are most likely DEG/ENaC, but not GdCl_3_-sensitive stretch-activated cation channels, such as the TRP and PIEZO family proteins^2,37^. The *C*. *elegans* genome encodes 32 DEG/ENaC genes. Among these, three DEG/ENaC channel subunits, DEG-1, MEC-4, and MEC-10, have been identified as mechanosensory receptors^3,38–40^. Some other DEG/ENaC subunits, such as UNC-8, UNC-105, DEL-1, and DEGT-1, have also been implicated in mechanosensation^40–42^. Given the large number of DEG/ENaC genes present in *C*. *elegans*, the primary mechanosensory receptor in RnB neurons has yet to be identified.

Male RnA neurons are thought to be essential for contact responses, scanning, and turning, whereas RnB neurons are only crucial for contact responses^12^. Since most steps of the mating behavior involve direct male-hermaphrodite body contact, RnA neurons are speculated to play a more important role in mechanosensation than RnB neurons^12^. Our data support the idea that RnA neurons may act as mechanosensory neurons. Nevertheless, we only got low efficiency on recording of touch-induced calcium responses in RnA neurons. TRP-4, a mechano-gated TRP channel, mediates touch sensation in CEP neurons and PDE neurons^4,25^, and it is expressed in some RnA neurons^12,24^. Surprisingly, our data hint that TRP-4 might not participate in mechanosensation in RnA neurons, consistent with previously reported observations that *trp-4* null mutants appear almost normal for all male mating sub-behaviors^12^. Our study suggests that male-specific neurons of *C*. *elegans* may provide an outstanding context for teasing out the molecular mechanisms of mechanosensation *in vivo*.

## Materials and Methods

### Strains

*C*. *elegans* strains were maintained under standard conditions^43^. Well-fed day 1 adult male were used in all experiments. The strains used in this study are as follows: Bristol N2 (*Caenorhabditis* Genetics Center), ST693 (*him-5(e1409); kanIs5[pkd-2::mCherry* + *pkd-2::GCaMP5*.*0* + *odr-1::DsRed]*), ST677 (*him-5(e1409); kanIs6[tba-9::mCherry* + *tba-9::GCaMP5*.*0]*), ST1295 (*him-5(e1409); unc-13(e51); kanIs5*), ST781 (*him-5(e1409); eat-4(ky5); kanIs5*), ST1298 (*him-5(e1409); unc-31(e928); kanIs5*), ST699 (*him-5(e1409); pkd-2(sy606); kanIs5*), ST704 (*him-5(e1409); pkd-2(sy606); lov-1(sy582); kanIs5*), ST767 (*him-5(e1409); osm-9(ky10); kanIs5*). Strains carried *him-5(e1409)* mutation could generate high incidence of males.

### Calcium Imaging

Individual animals were glued on a coverglass using a cyanoacrylate-based glue (Gluture Topical Tissue Adhesive, Abbott Laboratories) and immersed in bath solution (145 mM NaCl, 2.5 mM KCl, 1 mM MgCl_2_, 5 mM CaCl_2_, 10 mM HEPES, 20 mM glucose, pH adjusted to 7.3 with NaOH). The calcium indicator GCaMP5.0 was used to measure the intracellular calcium signals^22,44,45^. Imaging was acquired on an Olympus microscope (BX51WI) with a 60× objective lens. Raw image data were acquired with an Andor DL-604M EMCCD camera and micro-Manager 1.4 software. GCaMP5.0 was excited by a Lambda XL light source and fluorescent signals were collected at a rate of 1 Hz. The average GCaMP5.0 signal from the first 10 s before stimulus was taken as F0, and ΔF/F0 was calculated for each data point. The data was analyzed using Image J.

### Mechanical Stimulation

Touch stimulation was delivered to the cell using a tip diameter of ~1 μm borosilicate glass capillary driven by a piezoelectric actuator (PI) mounted on a micromanipulator (Sutter)^43^. The needle was placed perpendicular to the worm’s tail. In the “on” phase, the needle was moved toward the worm’s tail so that it could probe into the worm’s tail on the cilia of the ray neurons and then held on the cilia for 500 ms. In the “OFF” phase the needle was returned to its original position.

### Statistical Analysis

Data analysis was performed using GraphPad Prism 6 software. Error bars were mean ± SEM. N represents the number of cells. P values were determined by Student’s t test. P < 0.05 was regarded as statistically significant.

## Electronic supplementary material

Supplemental information

Movie S1

## References

[1] Katta S, Krieg M, Goodman MB (2015). Feeling force: physical and physiological principles enabling sensory mechanotransduction. Annu Rev Cell Dev Biol.

[2] Delmas P, Coste B (2013). Mechano-gated ion channels in sensory systems. Cell.

[3] Geffeney SL (2011). DEG/ENaC but not TRP channels are the major mechanoelectrical transduction channels in a C. elegans nociceptor. Neuron.

[4] Kang L, Gao J, Schafer WR, Xie Z, Xu XZ (2010). C. elegans TRP family protein TRP-4 is a pore-forming subunit of a native mechanotransduction channel. Neuron.

[5] Yan Z (2013). Drosophila NOMPC is a mechanotransduction channel subunit for gentle-touch sensation. Nature.

[6] Coste B (2012). Piezo proteins are pore-forming subunits of mechanically activated channels. Nature.

[7] Wu Z, Muller U (2016). Molecular Identity of the Mechanotransduction Channel in Hair Cells: Not Quiet There Yet. J Neurosci.

[8] Corey DP, Holt JR (2016). Are TMCs the Mechanotransduction Channels of Vertebrate Hair Cells?. J Neurosci.

[9] Christensen AP, Corey DP (2007). TRP channels in mechanosensation: direct or indirect activation?. Nat Rev Neurosci.

[10] Corsi AK, Wightman B, Chalfie M (2015). A Transparent Window into Biology: A Primer on Caenorhabditis elegans. WormBook.

[11] Barr, M. M. & Garcia, L. R. Male mating behavior. *WormBook*, 1–11, 10.1895/wormbook.1.78.1 (2006). 10.1895/wormbook.1.78.1 PMC4780960 18050467

[12] Koo PK, Bian X, Sherlekar AL, Bunkers MR, Lints R (2011). The robustness of Caenorhabditis elegans male mating behavior depends on the distributed properties of ray sensory neurons and their output through core and male-specific targets. J Neurosci.

[13] Sulston JE, Albertson DG, Thomson JN (1980). The Caenorhabditis elegans male: postembryonic development of nongonadal structures. Dev Biol.

[14] Sulston JE, Horvitz HR (1977). Post-embryonic cell lineages of the nematode, Caenorhabditis elegans. Dev Biol.

[15] Liu KS, Sternberg PW (1995). Sensory regulation of male mating behavior in Caenorhabditis elegans. Neuron.

[16] Barr MM (2001). The Caenorhabditis elegans autosomal dominant polycystic kidney disease gene homologs lov-1 and pkd-2 act in the same pathway. Curr Biol.

[17] Barr MM, Sternberg PW (1999). A polycystic kidney-disease gene homologue required for male mating behaviour in C. elegans. Nature.

[18] Praetorius HA, Spring KR (2005). A physiological view of the primary cilium. Annu Rev Physiol.

[19] Patel A (2015). The primary cilium calcium channels and their role in flow sensing. Pflugers Arch.

[20] DeCaen PG, Delling M, Vien TN, Clapham DE (2013). Direct recording and molecular identification of the calcium channel of primary cilia. Nature.

[21] Bae YK (2006). General and cell-type specific mechanisms target TRPP2/PKD-2 to cilia. Development.

[22] Akerboom J (2012). Optimization of a GCaMP calcium indicator for neural activity imaging. J Neurosci.

[23] Hurd DD, Miller RM, Nunez L, Portman DS (2010). Specific alpha- and beta-tubulin isotypes optimize the functions of sensory Cilia in Caenorhabditis elegans. Genetics.

[24] Li W, Feng Z, Sternberg PW, Xu XZAC (2006). elegans stretch receptor neuron revealed by a mechanosensitive TRP channel homologue. Nature.

[25] Li W, Kang L, Piggott BJ, Feng Z, Xu XZ (2011). The neural circuits and sensory channels mediating harsh touch sensation in Caenorhabditis elegans. Nature communications.

[26] Yang X (2015). Syntaxin opening by the MUN domain underlies the function of Munc13 in synaptic-vesicle priming. Nat Struct Mol Biol.

[27] Zhou KM (2007). PKA activation bypasses the requirement for UNC-31 in the docking of dense core vesicles from C. elegans neurons. Neuron.

[28] Lee RY, Sawin ER, Chalfie M, Horvitz HR, Avery L (1999). EAT-4, a homolog of a mammalian sodium-dependent inorganic phosphate cotransporter, is necessary for glutamatergic neurotransmission in caenorhabditis elegans. J Neurosci.

[29] Colbert HA, Smith TL, Bargmann CI (1997). OSM-9, a novel protein with structural similarity to channels, is required for olfaction, mechanosensation, and olfactory adaptation in Caenorhabditis elegans. The Journal of neuroscience: the official journal of the Society for Neuroscience.

[30] Knobel KM, Peden EM, Barr MM (2008). Distinct protein domains regulate ciliary targeting and function of C. elegans PKD-2. Exp Cell Res.

[31] White JQ (2007). The sensory circuitry for sexual attraction in C. elegans males. Curr Biol.

[32] Li GR, Baumgarten CM (2001). Modulation of cardiac Na(+) current by gadolinium, a blocker of stretch-induced arrhythmias. Am J Physiol Heart Circ Physiol.

[33] Goodman, M. B. Mechanosensation. *WormBook*, 1–14, 10.1895/wormbook.1.62.1 (2006). 10.1895/wormbook.1.62.1 PMC2806189 18050466

[34] Zhang W (2015). Ankyrin Repeats Convey Force to Gate the NOMPC Mechanotransduction Channel. Cell.

[35] Schuler A (2015). The Rise and Fall of TRP-N, an Ancient Family of Mechanogated Ion Channels, in Metazoa. Genome Biol Evol.

[36] Wu X (2016). Hair-Cell Mechanotransduction Persists in TRP Channel Knockout Mice. PLoS One.

[37] Wu J, Lewis AH, Grandl J (2017). Touch, Tension, and Transduction - The Function and Regulation of Piezo Ion Channels. Trends Biochem Sci.

[38] O’Hagan R, Chalfie M, Goodman MB (2005). The MEC-4 DEG/ENaC channel of Caenorhabditis elegans touch receptor neurons transduces mechanical signals. Nat Neurosci.

[39] Arnadottir J (2011). The DEG/ENaC protein MEC-10 regulates the transduction channel complex in Caenorhabditis elegans touch receptor neurons. J Neurosci.

[40] Hobert, O. The neuronal genome of Caenorhabditis elegans. *WormBook*, 1–106, 10.1895/wormbook.1.161.1 (2013). 10.1895/wormbook.1.161.1 PMC4781646 24081909

[41] Bianchi L (2007). Mechanotransduction: touch and feel at the molecular level as modeled in Caenorhabditis elegans. Mol Neurobiol.

[42] Chatzigeorgiou M (2010). Specific roles for DEG/ENaC and TRP channels in touch and thermosensation in C. elegans nociceptors. Nat Neurosci.

[43] White JG, Southgate E, Thomson JN, Brenner S (1986). The structure of the nervous system of the nematode Caenorhabditis elegans. Philosophical transactions of the Royal Society of London. Series B, Biological sciences.

[44] Zou W (2017). Polymodal Responses in C. elegans Phasmid Neurons Rely on Multiple Intracellular and Intercellular Signaling Pathways. Sci Rep.

[45] Zhou, W. e*t al*. Ultrasound neuro-modulation chip: activation of sensory neurons in Caenorhabditis elegans by surface acoustic waves. *Lab Chip*, 10.1039/c7lc00163k (2017). 10.1039/c7lc00163k 28447086

