# Supplementary material for: OSM-9 and an amiloride-sensitive channel, but not PKD-2, are involved in mechanosensation in C. elegans male ray neurons
Source: Sci Rep. 2018 May 8;8:7192. doi: 10.1038/s41598-018-25542-1 (PMC5940728; doi:10.1038/s41598-018-25542-1)
Supplement: Supplementary file 1 — Supplemental information [file 41598_2018_25542_MOESM1_ESM.docx]

**OSM-9 and an amiloride-sensitive channel, but not PKD-2, are involved in mechanosensation in *C. elegans* male ray neurons**

Hu Zhang^1*^, Xiaomin Yue^1*^, Hankui Cheng^1*^, Xiaoyan Zhang^1^, Yang Cai^1,2^, Wenjuan Zou^1^, Guifang Huang^1^, Lufeng Cheng^2^, Fang Ye^3†^, Lijun Kang^1†^

1 Department of Neurobiology, Institute of Neuroscience, Key Laboratory of Medical Neurobiology of the Ministry of Health of China, Zhejiang University School of Medicine, Hangzhou, China.

2 Department of Pharmacology, Basic Medical College, Xinjiang Medical University Urumqi, China.

3 Department of Immunology, School of Preclinical Medicine, Guangxi Medical University, Nanning, China.

*These authors contributed equally to this work.

†Corresponding author: Fang Ye, 22 Shuangyong Rd., Nanning, Guangxi, 530021, P. R. China, Tel: (86)0771-5358212, Email: [yefang@gxmu.edu.cn](mailto:yefang@gxmu.edu.cn)

†Corresponding author: Lijun Kang, 866 Yu Hang Tang Rd., Hangzhou, Zhejiang 310058, P. R. China, Tel: (86)571-88208487, Fax: (86)571-88208487, Email: [kanglijun@zju.edu.cn](mailto:kanglijun@zju.edu.cn)

**Supplemental Figure**

**
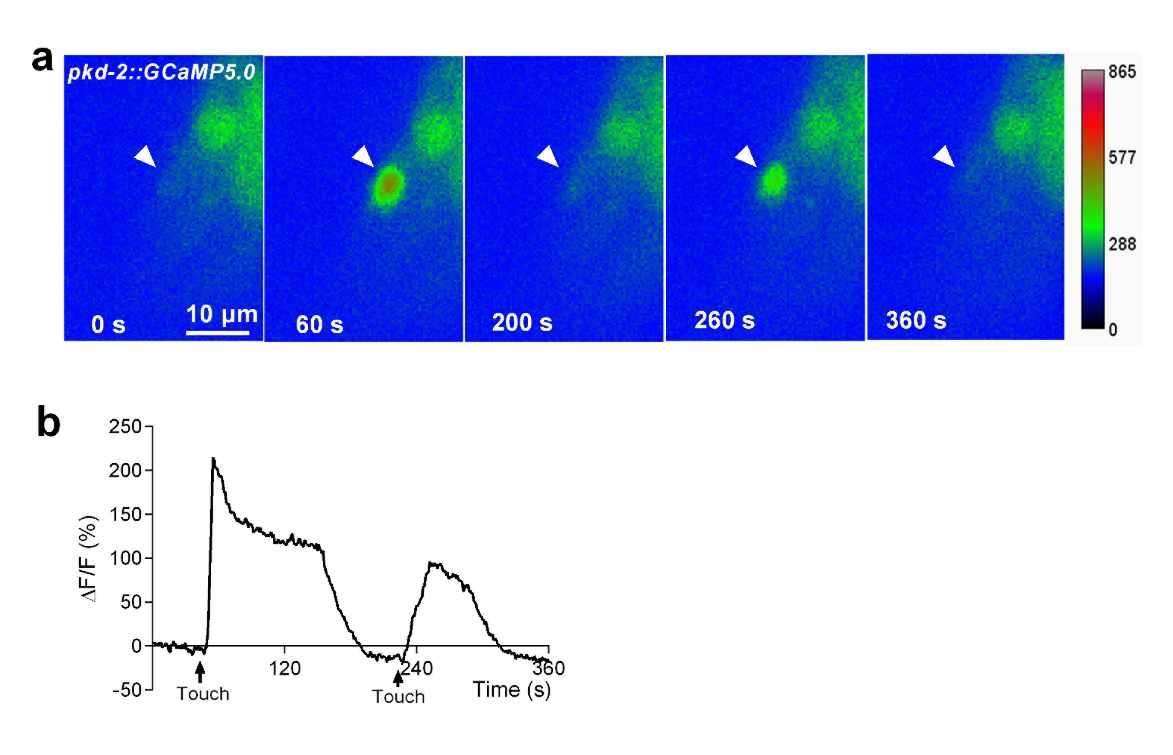
**

**Figure S1.** Representative time-lapse rainbow images of GCaMP5.0 based calcium responses (a) and soma fluorescence change (b) from a ray B neuron in Movie S1 induced by two successive mechanical stimuli of 15 μm displacement at the position of rays 1-3. The stimuli were delivered at 40 s and 220 s, respectively. Relative to Movie S1 and Figure 1.

**Supplemental Movie**

**Movie S1.** Rainbow images of GCaMP5.0 based calcium responses of a ray B neuron induced by two successive mechanical stimuli of 15 μm displacement at the position of rays 1-3. The stimuli were delivered at 40 s and 220 s, respectively.
